# Revisiting the putative TCR Cα dimerization model through structural analysis

**DOI:** 10.3389/fimmu.2013.00016

**Published:** 2013-01-30

**Authors:** Jia-Huai Wang, Ellis L. Reinherz

**Affiliations:** ^1^Laboratory of Immunobiology, Department of Medical Oncology, Dana-Farber Cancer Institute, Harvard Medical SchoolBoston, MA, USA; ^2^Department of Pediatrics, Harvard Medical SchoolBoston, MA, USA; ^3^Department of Biological Chemistry and Molecular Pharmacology, Harvard Medical SchoolBoston, MA, USA; ^4^Department of Medicine, Harvard Medical SchoolBoston, MA, USA

**Keywords:** TCR, receptor dimerization, signal transduction, structural immunology

## Abstract

Despite major advances in T cell receptor (TCR) biology and structure, how peptide–MHC complex (pMHC) ligands trigger αβ TCR activation remains unresolved. Two views exist. One model postulates that monomeric TCR–pMHC ligation events are sufficient while a second proposes that TCR–TCR dimerization in *cis* via Cα domain interaction plus pMHC binding is critical. We scrutinized 22 known TCR/pMHC complex crystal structures, and did not find any predicted molecular Cα–Cα contacts in these crystals that would allow for physiological TCR dimerization. Moreover, the presence of conserved glycan adducts on the outer face of the Cα domain preclude the hypothesized TCR dimerization through the Cα domain. Observed functional consequences of Cα mutations are likely indirect, with TCR microclusters at the immunological synapse driven by TCR transmembrane/cytoplasmic interactions via signaling molecules, scaffold proteins, and/or cytoskeletal elements.

## BACKGROUND

αβ T lymphocytes are components of the adaptive immune system that allow vertebrates to distinguish abnormal or foreign cells from normal cells. This ‘‘self’’ versus ‘‘non-self’’ discrimination is endowed by surface-bound αβ T cell receptors (TCRs) that are selected in the thymus (reviewed in Rudolph et al., 2006; Smith-Garvin et al., 2009; Kim et al., 2012; Kuhns and Davis, 2012; Wang and Reinherz, 2012). In vertebrates, there are millions to billions of αβ T cells, each with a slightly different TCR structure on their surface that confers a unique antigen-binding specificity. TCRs recognize antigens bound to MHC molecules on the surface of other cells. MHC molecules display an array of antigen peptides, providing a snapshot of the cell’s internal composition. Aberrant cellular processes, such as viral infection or oncogenic transformation, are reflected by alterations in antigen display. When a T cell senses a variant peptide (one derived from a viral protein, for example), cellular signaling pathways are initiated that cause the T cell to proliferate, differentiate, and mediate effector and regulatory functions. T cells are able to detect a variant peptide even if just a few copies are present among the hundreds of thousands of normal self-peptides that are displayed by the cell-surface MHC molecules.

The remarkable specificity and sensitivity at the heart of protective T cell immunity has provided the impetus for detailed cellular, biochemical, molecular, and structural studies of the TCR. The αβ TCR is a multimeric transmembrane (TM) complex composed of an Fab-like disulfide-linked antigen binding clonotypic heterodimer in non-covalent association with the signal transducing CD3 subunits (CD3εγ, CD3εδ, and CD3ζζ; dimer stoichiometry 1:1:1:1; Rudolph et al., 2006; Smith-Garvin et al., 2009; Kim et al., 2012; Kuhns and Davis, 2012; Wang and Reinherz, 2012). The α and β subunit ectodomains are composed of membrane distal Vα and Vβ variable domains linked to membrane proximal Cα and Cβ constant domains, respectively. These two constant domains are tethered to their individual TM segments via connecting peptides. Each CD3ε, γ, and δ subunit contains an extracellular immunoglobulin (Ig)-like domain, a membrane-proximal stalk region, a TM segment and a cytoplasmic tail. The interaction between an αβ TCR heterodimer on the T cell and a peptide–MHC complex (pMHC) ligand on an antigen-presenting cell (APC) initiates a cascade of downstream signaling events. These events are transmitted via the immunoreceptor tyrosine-based activation motif (ITAM) elements in the cytoplasmic tails of the associated CD3 subunits, whose lengths are substantial relative to those of the TCR α and β tails and couple with critical tyrosine kinase pathways involving lck and Zap70 (Reth, 1989; Letourneur and Klausner, 1992; Acuto et al., 2008; Au-Yueng et al., 2009; van der Merwe and Dushek, 2011).

## ELUCIDATION OF TM RECEPTOR SIGNALING IN OTHER SYSTEMS: POTENTIAL RELEVANCE FOR THE TCR MECHANISM

Transmembrane signaling is one of the most intriguing and fundamental topics in cell biology. The receptor component of a TM protein functions to receive an environmental message whereas the TM and cytoplasmic segments of the protein transduce the signal into the cell and onward to the nucleus, activating relevant genes and permitting adaptation to environmental changes. There has been tremendous progress in how receptors recognize ligands at the cell surface and the elucidation of the various signaling cascades transmitting information inside the cell. For the growth hormone receptor (GHR), a member of the cytokine receptor superfamily, binding of the growth hormone ligand causes two GHRs to dimerize at the cell surface (Cunningham et al., 1991; De Vos et al., 1992). In so doing, these GHRs bring their two cytoplasmic tails in proximity to mediate cross-phosphorylation inside the cell, resulting in activation. In the case of members of the tyrosine kinase receptor family such as epithelial growth factor receptor (EGFR), two EGF ligands bind to two EGFRs (Schlessinger, 2002). Receptor oligomerization appears to be one general mechanism for mediating signal transduction. Of note, the juxtaposition of receptors in an oligomer must be precise, as not any dimer will suffice to initiate signaling (Ballinger and Wells, 1998). On the other hand, analysis of G protein-coupled receptors (GPCR) show how allosteric changes in the TM segments of one receptor modulate TM signaling without a requirement for receptor oligomerization (Rasmussen et al., 2011).

Despite a wealth of structural and functional data involving interactions between the TCR and antigenic peptides presented by MHC molecules on the cell surface as well as signaling events within the cell, it is still largely unclear how the engagement of the TCR by pMHC leads to subsequent activation of the intracellular machinery. Given the important role of oligomerization in cytokine and tyrosine kinase receptor superfamilies, the possibility of TCR oligomerization as a signaling modality has been considered early on in the field of T cell biology. In this regard, anticlonotypic TCR αβ mAbs or anti-CD3ε mAb, linked to solid supports were found to be stimulatory for T cells, replacing the requirement for both peptide and MHC on APCs in the activation process (Meuer et al., 1983, 1984). In contrast, soluble antibody or Fab fragments of those antibodies were non-activating. Likewise, more recently, TCR signal transduction initiation was found to require engagements of multiple pMHC ligands; pMHC monomer were non-stimulatory and pMHC oligomers were efficient activators (Boniface et al., 1998; Krogsgaard et al., 2005). These studies were interpreted as showing that ligand-driven formation of TCR clusters is required for effective activation, accounting for T cell specificity and sensitivity.

Along these lines, ligand-specific oligomerization of αβ TCR heterodimeric ectodomains was demonstrated in solution using light scattering methodology (Reich et al., 1997). It was reported that in the presence of specific agonist pMHC, that TCR/pMHC complexes underwent oligomerization. Surface plasma resonance studies followed, revealing biphasic binding kinetics at 37°C, interpreted via a model of TCR dimerization (Alam et al., 1999). However, another study using these same methods, as well as sedimentation equilibrium analytic ultracentrifugation, in addition, failed to replicate those findings either with class I or class II MHC restricted TCRs and their physiologic pMHC ligands (Baker and Wiley, 2001). In view of these inconsistencies, the role played by TCR oligomerization was studied further in the membrane context with fully assembled TCRs. Only recently have alternative force-transduction mechanisms been offered to explain the apparent inability of univalent ligands to trigger T cell responses (vide infra; Kim et al., 2012). Moreover, due to the paucity of structural information on TCR TM segments, any potential conformational changes therein upon ligand binding are unrecognized at present.

## MODELS OF TCR ECTODOMAIN TOPOLOGY

**FIGURE 1:**
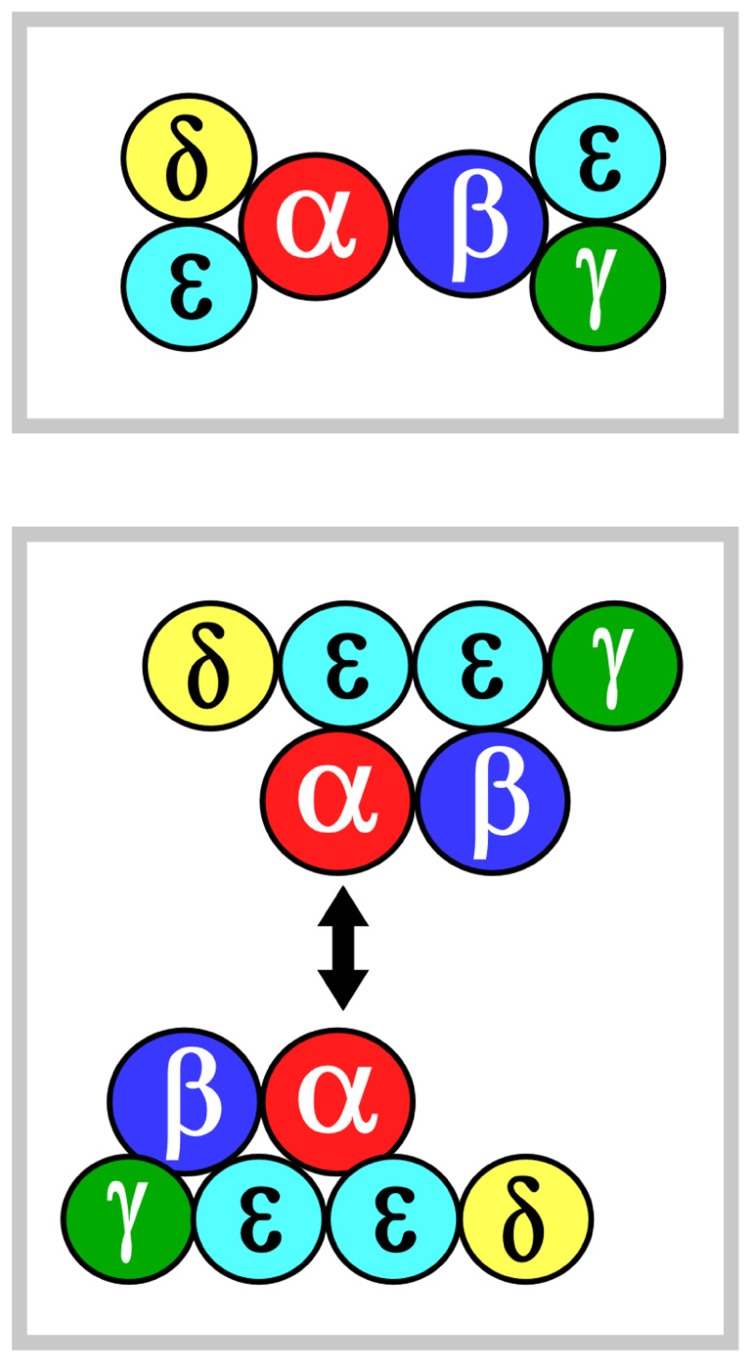
**αβ TCR complex subunit ectodomain topology**. Two models of the topology are shown in the top and bottom panels, termed models 1 and 2, respectively. These are viewed from above the T cell looking down. Color coding for the CD3 components are green, yellow and mint for CD3γ, CD3δ, and CD3ε, respectively. TCRα is in red and TCRβ is in blue. The CD3ζζ homodimer is omitted since it lacks an ectodomain structure. In model 2, the two-headed arrow is meant to indicate that Cα–Cα interaction dimerizes two TCR complexes (Kuhns et al., 2010). See the text for details.

Two models of TCR ectodomain topology on the T cell membrane are currently under consideration (Fernandes et al., 2012; Kim et al., 2012; Kuhns and Davis, 2012). Potential for αβ heterodimeric oligomerization differs in these two views. In both models, the αβ heterodimer is centrally positioned. **Figure 1** shows that in model 1 (top panel), CD3εδ and CD3εγ laterally flank α and β subunits, respectively. The rationale for this topology has been detailed extensively in the past and will not be reviewed here (Kim et al., 2012). In model 2 (bottom panel), the CD3 heterodimers localize to one face of the αβ heterodimer and CD3 εδ is rotated ~270° clockwise and CD3εγ rotated ~90° counterclockwise relative to model 1. This orientation juxtaposes the two non-glycosylated CD3 ectodomains and, thereby, allows the other side of TCR αβ to be available for homotypic dimerization. This ‘‘functional sidedness’’ was inferred by utilization of a dimerization reporter system and BaF3 pro-B cell transfection studies based on erythropoietin receptor (EPOR) signaling measuring BaF3 cell proliferation and CD3–EPOR fusion constructs (Kuhns et al., 2010; Kuhns and Davis, 2012). Similarly, a TCR α–EPOR chimera in conjunction with TCR β TM (TM derived from EPOR) was interpreted as offering functional evidence for Cα–Cα dimers (**Figure 1**, model 2). By mutation analysis in BaF3 cells, the dimer interface was mapped to the C and F strands of the Cα domain. In this view, the dimer juxtaposes two TCR complexes to facilitate signaling through the cellular membrane in an, as yet, undefined manner. Given this interesting set of results, we have carefully surveyed TCR/pMHC complex crystallographic data searching for structural evidence consistent with model 2.

## SURVEY OF MOLECULAR CONTACTS IN AVAILABLE CRYSTAL STRUCTURES DOES NOT SUPPORT THE TCR Cα–Cα DIMERIZATION MODEL

Protein–protein interactions in a living system and in a protein crystal obey the same physico-chemical rule in seeking an energy minimum. Not surprisingly, there have been numerous examples of protein–protein interactions observed in crystal structures reflecting physiologically relevant interactions in cellular systems. The field of structural immunology has served to unravel key aspects of immune function. Over the last 16 years, complex structures of TCR/pMHC, co-receptor/pMHC, and the ternary complex of TCR/pMHC/CD4 derived from crystal structures have substantively advanced the field of immunology and opened new avenues for cellular and molecular functional studies (reviewed in Rudolph et al., 2006; Smith-Garvin et al., 2009; Kim et al., 2012; Kuhns and Davis, 2012; Wang and Reinherz, 2012; Yin et al., 2012). Occasionally, crystal packing patterns may suggest a misleading conclusion but that can be assessed through further structural analyses and vetted by mutational studies directed at crystallographically identified contact sites to ensure their biological relevance.

We then assert that if a TCR, in fact, dimerizes through its Cα domain on the T cell surface, one should observe such a dimer in at least some TCR/pHMC crystal lattices. Based on this assumption, we set out to test the proposed TCR dimer model by scrutinizing available TCR/pMHC crystal structures. **Table 1** lists 22 crystal structures of TCRs in complex with pMHCs. These cover both peptide-bound class I and class II MHC molecules from humans and mice including one ternary complex of a TCR/pMHC/CD4 deposited in the protein data bank (PDB). Only those structures containing a TCR Cα domain were included in this survey. The molecular packing pattern in the crystal for each of these structures listed in **Table 1** has been generated and displayed via computer graphics. Specifically, potential interactions involving the Cα domain in the crystals were scrutinized. Interestingly, only three structures manifest Cα–Cα interaction (PDB codes 2PYF, 3FFC, and 3MBE). In the remaining 19 crystal structures, the TCR Cα domain either contacts the TCR β chain or the MHC molecule or remains unengaged in molecular interactions with other molecules.

**Table 1:** Molecular contacts among complexes in human and murine αβ TCR/pMHC crystals.

PDB file	MHC class	Species	Cα contact
1BD2	I	Human	Cα contacts TCR Vβ
1FYT	II	Human	Cα contacts MHCII β2
1J8H	II	Human	Cα contacts MHCII β2
1MI5	I	Human	Cα contacts TCR Vβ
1OGA	I	Human	Cα contacts TCR Vβ
1QSE	I	Human	Cα contacts MHCI β1
1ZGL	II	Human	Four molecules. Two Cα contact TCR Vβ, the other MHCII β2
2AK4	I	Human	Four molecules. Two Cα contact MHCI α3/β2, the other α3
2CKB	I	Mouse	Cα contacts the elbow of TCR Vβ–Cβ
2IAM	II	Human	Cα contacts MHCII β2
**2PYF**	I	Human	Cα contacts TCR Cα and Cβ
2WBJ	II	Human	Two molecules. One Cα contacts MHCII α2, the other has no contact
3C5Z	II	Mouse	Two molecules. One Cα contacts TCR Vβ, the other Cβ
3C6O	II	Mouse	Two molecules. One Cα contacts MHC α2, and the other Vβ–Cβ
**3FFC**	I	Human	Cα forms dimer. FG loop and G strand are involved
3HG1	I	Human	Cα barely contacts Vβ
**3MBE**	II	Mouse	Cα forms dimer. F and G strands are involved
3PL6	II	Human	Cα contacts MHCII β2
3RDT	II	Mouse	Cα contacts MHCII α2
3RGV	I	Mouse	Cα contacts MHCI peptide-binding domains
3SJV	I	Human	Four molecules. All Cα contact the MHCI peptide-binding domain
3TOE	II	Human	Cα contacts CD4 only (this is a TCR/pMHC/CD4 complex)

*PDB file numbers showing any Cα–Cα contacts are highlighted in bold.*

**FIGURE 2:**
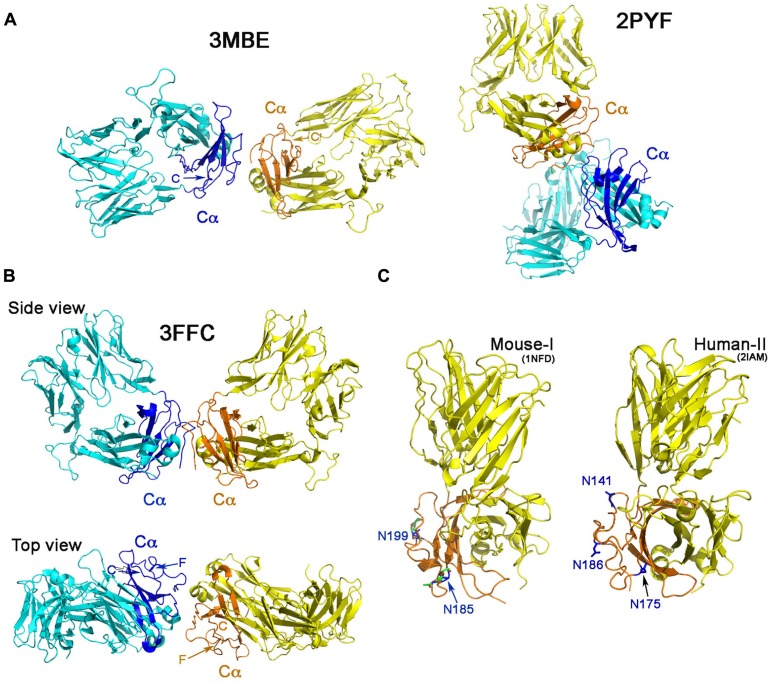
**‘‘Dimers’’ involving the Cα domain in αβ TCR/pMHC complexes**. **(A)** The dimeric model in two crystal structures. The structure of 2PYF in the right panel is not a symmetric dimer, whereas the structure of 3MBE in the left panel reveals a dimer apparently lying flat on the cell membrane, so of questionable biological significance. In panels **(A)** and **(B)** the Cα domains are shown in dark blue and orange. **(B)** Two views of the structure of 3FFC show a possible dimer standing on the plasma membrane. From the top view, it is apparent that neither C strand, nor F strand participates in dimer formation. **(C)** The potential N-linked glycosylation sites in mouse (left panel) and human (right panel) are labeled with asparagines in blue color. These sites are all at the outer face of the Cα domain.

**Figure 2A** shows the Cα–Cα interactions in structures 2PYF and 3MBE. In 2PYF, the manner in which two Cα domains contact one another cannot be regarded as forming a dimer. They barely touch, doing so in asymmetric fashion. On the other hand, the two Cα domains in the structure 3MBE do form a dimer, but that dimer would force the two TCR molecules to lie horizontally on the plasma membrane in a clearly unphysiologic mode. The structure 3FFC, depicted in two views in **Figure 2B** appears to present the best possible dimer. The side view illustrates two TCR heterodimers standing side by side with their C-termini pointed toward the membrane at the bottom. The top view orientation is looking down from above. In this perspective, it is obvious that neither F strand nor C strand of the Cα domain is located at the interface. Thus, although the dimer in this one crystal might mediate a Cα–Cα interaction of potential physiologic significance, it is not in agreement with the proposed TCR dimer model. The conclusion is very clear from the survey: there is no crystallographic evidence to support TCR dimerization through the C and F β strands on the Cα domain’s outer face inferred from earlier mutational study.

## POTENTIAL GLYCANS ON THE Cα DOMAIN PREVENT TCR DIMERIZATION AT ITS OUTER SURFACE

An additional argument against the proposed TCR dimer model is the fact that there are potential glycosylation sites located on the outer face of the TCR Cα domain (**Figure 2C**). It is known that almost all immune receptors are glycosylated (Rudd et al., 2001). In fact, the most common post-translational modification of these cell surface receptors is the *N*-linked glycosylation on Asn in the N-X-S/T sequon, where X represents any kind of amino acid in the motif. The glycan has GlcNAc_2_Man_3_ as its core attached to Asn. Many carbohydrate residues then further branch out from the two forked mannoses to form a rather long (more than a dozen residues) oligosaccharide adduct, significantly extending away from the protein surface. Functionally, the glycans help to orient ligand-binding surfaces, impact lateral mobility of receptors, protect receptors from the attack by protease and also restrict non-functional protein–protein interactions (Rudd et al., 2001). Published crystal structures of these receptors when expressed in eukaryotic systems, usually reveal the sugar moieties linked to Asn residue at those potential glycosylation sites. In our early work of the mouse class I restricted TCR, N15, the protein was expressed in the CHO Lec 3.2.8.1 system and subsequently treated with Endo-H. One sugar moiety left at each of the potential *N*-linked glycosylation sites was well defined in the electron density in the crystal structure (PDB code 1NFD; Wang et al., 1998) demonstrating that these sites are utilized in the TCR. The two sites at Asn185 and Asn199 on the murine Cα domain are shown in **Figure 2C**, the left panel. Depicted on the right panel of **Figure 2C** is the human class II restricted TCR (expressed in *E. coli*), E8 (PDB code 2IAM). Three potential glycosylation sites on its Cα domain are shown at Asn141, Asn175, and Asn186. The TCR C module is conserved within a particular species on all TCRs. As shown in the mouse, the two glycans are at the Ig-like domain’s EF loop (N185 in 1NFD) and the FG loop (N189 in 1NFD). For the human (in 2IAM), the three glycans are positioned at the beginning of C strand (N141), EF loop (N175), and on the F strand (N186). The crystal structure, however, did not show any glycans attached to the sites since the protein was expressed in *E. coli.* According to the rules reported from a systematic study (Kasturi et al., 1997), for a sequon of N-X-S, as long as the X is not Trp, Asp, Glu, or Leu, the site should be efficiently glycosylated. For the human TCR Cα domain, the three conserved potential sites are N141VS, N175KS, and N186NS. Hence, glycans are expected to exist on those sites, all located on the outer face of Cα domain. Most notable is the conserved site for glycan addition on the F strand in human TCR Cα domain. In the presence of the glycan adduct, it is not possible for this β strand to be involved in TCR homodimerization. Instead, this and the other Cα domain glycans likely prevents lateral protein–protein interaction and maintain the TCR αβ heterodimer upright on the cell surface as reviewed previously (Rudd et al., 2001).

## NEW INSIGHTS ON TCR BIOLOGY

A variety of recent experiments suggest that the TCR is a mechanosensor, converting mechanical energy into biochemical signals upon ligation (Kim et al., 2009; Li et al., 2010; Ma and Finkel, 2010; Husson et al., 2011; Judokusumo et al., 2012). Tangential force applied by optical tweezer technology using specific pMHC ligand-coated beads results in the αβ heterodimer exerting torque on the CD3 heterdimers as a consequence of molecular movement (Kim et al., 2009). Such force, being low piconewton in magnitude, is readily generated as T cells scan various epithelial or APC surfaces during immune surveillance via integrin-mediated adhesion events and prior to TCR-driven stop movement signals. At the immunological synapse, when cell migration has terminated, force continues to be exerted on the TCR via microcluster formation and retrograde actin-based trafficking from inside the cell (Yokosuka et al., 2008). Predicted alterations in TCR TM segments and surrounding lipid likely convert ectodo-main ligation into the earliest intracellular signaling events (Kim et al., 2012).

## CONCLUDING REMARKS

In summary, from our survey of TCR crystal structures, there is no evidence consistent with the proposed TCR dimer model among nearly two-dozen TCR/pMHC complex structures studied. More strikingly, the presence of bulky glycans on the outer face of TCR Cα domain, including the F strand in the human TCR, will prevent TCR dimerization there. Observed microcluster formation at the immunological synapse almost certainly results from interactions involving other TCR complex elements, including the cytoplas-mic tail.

## Conflict of Interest Statement

The authors declare that the research was conducted in the absence of any commercial or financial relationships that could be construed as a potential conflict of interest.
